# Mapping Drivers of Coronary Endothelial Activation and Endothelial‐to‐Mesenchymal Transition through Mimicking of Multimediator Inflammation in Kawasaki Disease Context

**DOI:** 10.1002/acr2.70110

**Published:** 2025-10-12

**Authors:** Pia Buthe, Marie Carlotta Limburg, Sabrina Fuehner, Julia Kuehn, André Jakob, Isabelle Koné‐Paut, Stéphanie Tellier, Alexandre Belot, Linda Rossi‐Semerano, Perrine Dusser‐Benesty, Isabelle Marie, Jana Merfort, Katja Masjosthusmann, Claas Hinze, Helmut Wittkowski, Dirk Foell, Christoph Kessel

**Affiliations:** ^1^ Department of Pediatric Rheumatology and Immunology University Children's Hospital Muenster Muenster Germany; ^2^ Department of Pediatric Cardiology and Pediatric Intensive Care Ludwig‐Maximilians‐University Munich Germany; ^3^ Division of Pediatric Rheumatology and CEREMAIA, Bicêtre Hospital, AP‐HP University of Paris Saclay Le Kremlin‐Bicêtre France; ^4^ Department of Pediatrics, Divisions of Nephrology, Rheumatology and Internal Medicine University of Toulouse Toulouse France; ^5^ Departments of Pediatrics, Division of Rheumatology, Dermatology and Nephrology University of Lyon Lyon France; ^6^ Department of General Pediatrics University Children's Hospital Muenster Muenster Germany

## Abstract

**Objective:**

Kawasaki disease (KD) is an acute systemic vasculitis predominantly affecting coronary arteries of infants and children. We recently identified leucin‐rich α‐2‐glycoprotein 1 (LRG‐1) as known transforming growth factor β1 (TGFβ1) signal‐modulating molecule, orchestrating endothelial activation and cardiac remodeling, as associated with interleukin‐1β (IL‐1β) signaling in KD. In the present study, we aimed to assess the role for LRG‐1 as part of a multimediator inflammatory environment as a possible direct mediator of human coronary artery endothelial activation.

**Methods:**

Human coronary artery endothelial cells (HCAECs) were treated with a blood inflammatory matrix, with or without targeted inhibition of several inflammatory mediators, including LRG‐1, and were analyzed for inflammatory activation or endothelial‐to‐mesenchymal transition (EndMT) on gene expression level. Proteomic profiling of the inflammatory matrix, treatment‐naïve KD (n = 11), or healthy control serum samples (n = 10) was performed by proximity extension assay (n = 184 markers) and Luminex.

**Results:**

Proteomic analysis of KD serum samples and the inflammatory matrix revealed elevation of 37 versus 50 inflammatory proteins, respectively, with 19 significantly up‐regulated markers shared. The HCAEC culture with the inflammatory matrix resulted in inflammatory endothelial activation, which was most efficiently abrogated by IL‐1 receptor type 1 (IL‐1R1) inhibition compared to all other tested drugs. Whereas inflammatory endothelial activation can also link to TGFβ‐driven EndMT, which was supported by respective signatures in our KD serum proteomics, we observed that in vitro inflammatory matrix–induced EndMT was partly impaired by both IL‐1R1 and tumor necrosis factor inhibition compared to other tested drugs.

**Conclusions:**

Collectively, our observations in the context of a multimediator inflammatory environment indicate a prominent role of a specific clinically relevant cytokine signaling axis in inflammatory coronary artery endothelial activation and EndMT in the context of KD.

## INTRODUCTION

Kawasaki disease (KD) is an acute systemic vasculitis of early childhood (younger than five years of age), primarily affecting medium‐sized arteries and coronary arteries, in particular. It is the leading cause of acquired heart disease in children in developed countries. Clinical features and immune activation patterns in KD overlap with systemic juvenile idiopathic arthritis and multisystem inflammatory syndrome in children, including risks of hyperinflammation such as macrophage activation syndrome.^1^, ^2^

Although KD's exact cause still remains unknown, genetic susceptibility is evident, particularly among Asian populations. Variants in immune‐related genes (eg, *CD40L*, *FCGR2A*, *MBL2*, *ITPKC*, and *TGFB2*) suggest a multifactorial immune basis.^2^, ^3^ The disease involves both innate and adaptive immune activation, with elevated levels of cytokines such as interleukin‐6 (IL‐6), tumor necrosis factor (TNF), IL‐17A, IL‐1 receptor antagonist (IL‐1Ra), and chemokines (eg, CXCL9/10)^4^, ^5^, ^6^ and immune cell infiltration in the coronary artery wall.^7^ Importantly, endothelial inflammation, fibrosis, and endothelial‐to‐mesenchymal transition (EndMT) contribute to vascular damage and aneurysm formation.^8^, ^9^, ^10^

The standard treatment, intravenous immunoglobulins (IVIGs), reduces coronary artery aneurysm (CAA) risk significantly. However, 10% to 20% of patients remain IVIG resistant, leading to higher complication rates.^1^ Rescue therapies such as TNF and IL‐6 blockade show mixed success,^11^, ^12^, ^13^ with concerns about new CAA formation in patients receiving IL‐6 receptor (IL‐6R) blockade.^11^ Recent clinical and experimental data highlight IL‐1 signaling as a key driver of KD pathophysiology.^2^

In the KAWAKINRA trial (Eudract number: 2014‐002715‐41, ClinicalTrials.gov NCT02390596), the IL‐1Ra anakinra showed promising efficacy and safety in IVIG‐resistant KD.^14^ In a subsequent biomarker analysis of this trial, we linked leucin‐rich α‐2‐glycoprotein 1 (LRG‐1) levels to IL‐1β activity, inflammation, and anakinra dose response.^5^ Other studies also report elevated LRG‐1 levels in acute KD,^15^, ^16^ particularly in patients with CAA or in serum exosomes.^17^ LRG‐1 is produced by hepatocytes and granulocytes in response to inflammation^18^, ^19^ and modulates transforming growth factor β (TGFβ) signaling, contributing to endothelial activation, neovascularization, and fibrosis.^20^, ^21^

Given these findings and the known impact of TGFβ pathway variants on KD outcomes,^22^ the present study combines advanced proteomics and functional assays to investigate the role of LRG‐1 compared to other inflammatory mediators in triggering EndMT and endothelial activation in human coronary artery endothelial cells (HCAECs), under conditions mimicking the inflammatory environment in KD.

## MATERIALS AND METHODS

All information related to study participants, sample collection, and applied methodology for proteomic analysis and in vitro assays is detailed in the Supplemental Methods section.

## RESULTS

### Serum proteomics in KD confirms multifactorial inflammation and indicates endothelial dysfunction

The etiology and specific triggers of KD are currently unknown. Vasculitis in KD involves both innate and adaptive immune cells, which is also reflected by the soluble inflammatory mediators that have been reported to circulate in elevated levels in the serum or plasma of patients with KD.^4^, ^5^, ^6^

**Figure 1:**
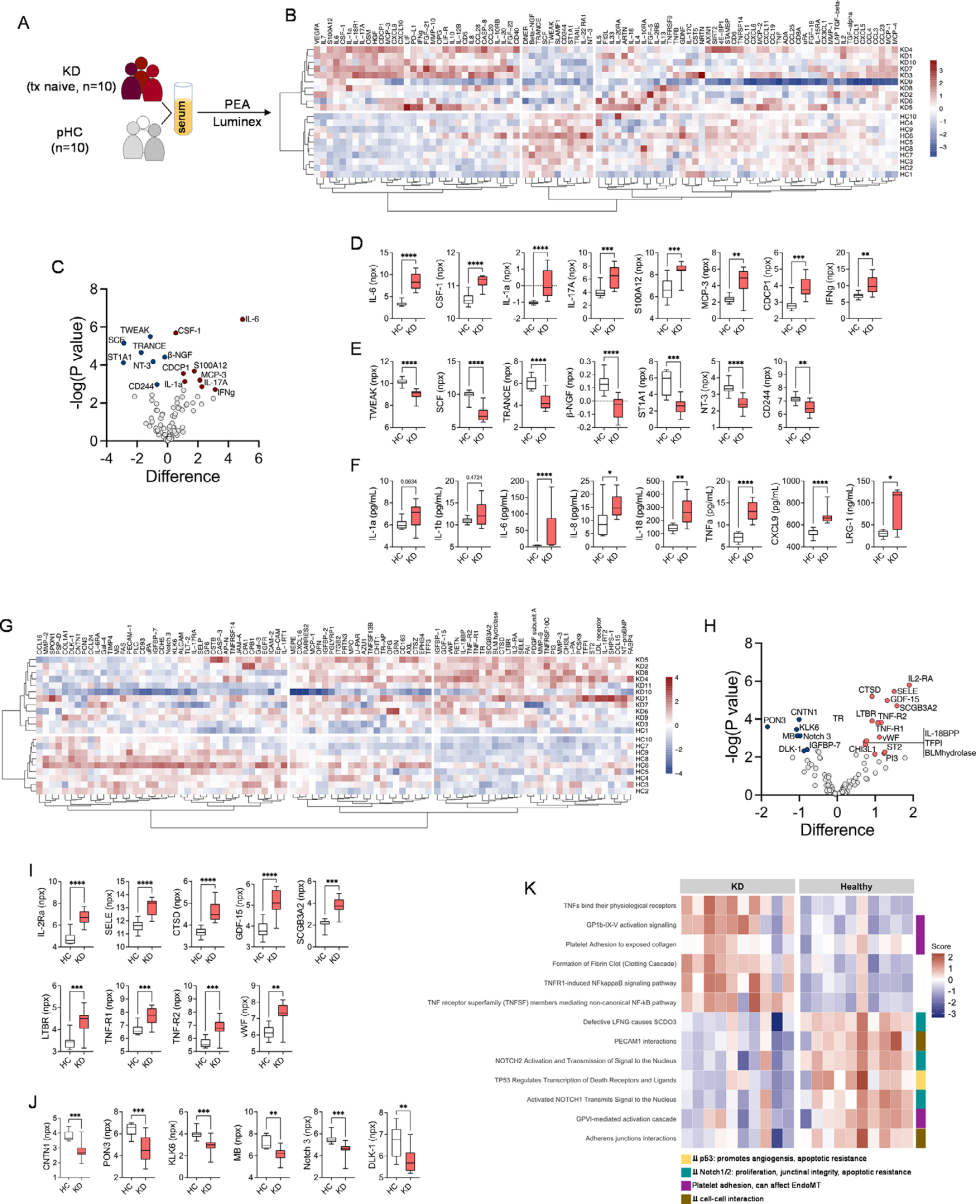
Inflammatory signature of serum samples from patients with KD and the blood inflammatory matrix. (A) Serum samples of treatment‐naïve patients with KD (n = 10) and HCs (n = 10) were analyzed using (B–E) PEA (Olink Target 96 Inflammation Panel). (B) The heat map depicts unsupervised clustering using correlation distance and ward. D2 linkage of npx data obtained from PEA. (C) The volcano plot highlights markers of inflammation significantly up‐ (red) or down‐regulated (blue) compared to npx data obtained from HCs. (D–F) Individual up‐ (D) and down‐regulated markers (E) in KD serum samples compared to HC according to PEA and (F) additional multiplexed bead array assay (Luminex) are shown. (D–F) Data were analyzed by the Mann‐Whitney U test. (G) Serum samples of treatment‐naïve patients with KD (n = 11) and HCs (n = 10) were analyzed by PEA (Olink Target 96 Cardiovascular Panel III). The heat map depicts unsupervised clustering using correlation distance and ward. D2 linkage of npx data obtained from PEA. (H) The volcano plot highlights cardiovascular serum markers significantly up‐ (red) or down‐regulated (blue) compared to npx data obtained from HCs. (I and J) Individual up‐ (I) and down‐regulated markers (J) in KD serum samples compared to HC according to PEA. Data were analyzed by Mann‐Whitney U test. (K) PathfindR analysis of PEA data identifies reactome signatures that—when down‐regulated as in KD—can promote angiogenesis, affect junctional (cell–cell contact) integrity, and platelet adhesion. *P < 0.05; **P < 0.01; ***P < 0.001; ****P < 0.0001. HC, healthy control; KD, Kawasaki disease; npx, normalized protein expression; PEA, proximity extension assay.

In our hands, proteomic profiling in treatment‐naïve patients with KD and healthy controls (HCs; Table 1) for serum markers of inflammation using proximity elongation assay (Figure 1A–E) and complementary Luminex (Figure 1F) revealed differential expression of several markers including LRG‐1 to separate treatment‐naïve patients with KD from HCs. When analyzing serum samples of untreated patients with KD and HCs (Table 1) for a broad panel of known and exploratory markers for human cardiovascular activation and inflammation (Figure 1G), we observed proteins implicated in endothelial cell activation and proliferation (SELE: E‐selectin; CHI3L1: YKL‐40), EndMT (GDF‐15: growth differentiation factor 15; CHI3L1: YKL‐40), fibrosis (ST2: soluble interleukin 1 receptor‐like 1; CTSD: cathepsin D) and arteriosclerosis (LTBR: lymphotoxin β receptor) among the top up‐regulated markers (Figure 1G–I; Table S3). Conversely, most of the observed down‐regulated markers are reported to be involved in cell adhesion (contactin 1), protection from arteriosclerosis (paraoxonase 3), and restriction of angiogenesis (insulin‐like growth factor‐binding protein 7; (Figure 1G, H, and J; Table S3). When subjecting the entire cardiovascular proteomic dataset to pathway enrichment analysis, this indicates TNF signaling, NF‐κB activation, and platelet adhesion and clotting processes as up‐regulated compared to HCs (Figure 1K). Potentially linking to endothelial dysfunction as in the focus of the present investigations, this analysis reveals decreased junctional integrity and cell–cell contact as well as enhanced proliferation, angiogenesis, and apoptotic resistance as prominent features of KD compared to healthy individuals (Figure 1K).

**Table 1:** Demographic data of study participants enrolled for serum proteomic analysis*

	HC^a^ (n = 10)	KD (n = 11)
Mean age, y	12.1	2.0
Age range, y	9.6–16.7	1.25–2.75
Sex	4f, 6m	5f, 6m
Disease features, % (n)^b^		
Fever	0 (0/10)	100 (11/11)^b^
Cardiovascular involvement (CA dilations or myocarditis)	n.r.	100 (11/11)
CAA	n.r.	9 (1/11)
Conjunctivitis	n.r.	100 (11/11)
Cutaneous symptoms (rash)	n.r.	100 (11/11)
Laboratory parameters (at sampling time point), median (IQR)		
CRP, mg/dL	<0.5	14.3 (11.3–21.0)
ESR, mm/hr	n.d.	105 (84–115)
Leucocytes, 1,000/μL	n.d.	15.9 (15.2–19.5)
Thrombocytes, 1,000/μL	n.d.	467 (314–678)
Treatment (at time of sampling)		
No treatment, % (n)^b^	100 (10/10)	100 (11/11)

* CA, coronary artery; CAA, coronary artery aneurysm, defined^1^ as z score of ≥ 2.5; CRP, C‐reactive protein; ESR, erythrocyte sedimentation rate; f, female; HC, healthy control; IQR, interquartile range; KD, Kawasaki disease; m, male; n.d., not determined; n.r., not recorded.

^a^ Noninflammatory controls (pediatric, adolescent, and adult donors).

^b^ Numbers in parentheses indicate positive cases of all cases in which this information was available.

### In multimediator inflammation, IL‐1, but not LRG‐1, is an important driver of inflammatory activation in HCAECs

Based on our previous findings,^5^ a primary objective of the present study was to investigate whether LRG‐1 could indeed mediate an inflammatory activation of HCAECs, when operating in concert with potent inflammatory mediators such as IL‐1β, TNFα, or IL‐6 as part of a multimediator inflammatory environment as in KD (Figure 1A–F). To investigate this, we set out for targeted inhibition of LRG‐1 using a novel monoclonal humanized therapeutic antibody (magacizumab). However, conducting such experiments directly in serum was significantly hampered by factors such as the small serum volumes obtainable from young patients (mean age: 2.7 years), the need for high‐quality serum without prior freeze–thawing cycles, and the requirement for samples from ideally treatment‐naïve patients with KD.

In such lines, using serum samples from patients with KD compared to serum samples from patients with sJIA and MIS‐C as controls (Table S1) revealed only a very modest effect on stimulation of HCAECs when assessed on gene expression level indicating inflammatory (*IL6* and *IL8*) or endothelial (*ICAM*] and *VCAM*) activation (Figure S1A). Interestingly, however, only KD serum stimulated *IL8* expression. Posttreatment KD serum (anakinra) induced less *IL8* expression, suggesting a therapy‐dependent reduction in endothelial activation, but overall effects were weak (Figure S1B).

To bypass limitations associated with experiments reliant on patients’ serum samples, we used a blood inflammatory matrix by stimulating healthy donor whole blood preparations ex vivo with lipopolysaccharide (LPS) and ATP. This could partially mimic KD‐like inflammatory stress, generating a cytokine‐rich environment for functional assays (Figure S2). Proximity elongation assay analysis of this matrix revealed elevation of 50 markers compared to unstimulated controls, 19 of which overlapped with elevated markers in KD serum. LRG‐1 was notably abundant, with concentrations rising from 174 ng/mL (unstimulated) to 364 ng/mL after stimulation (Figure S2).

**Figure 2:**
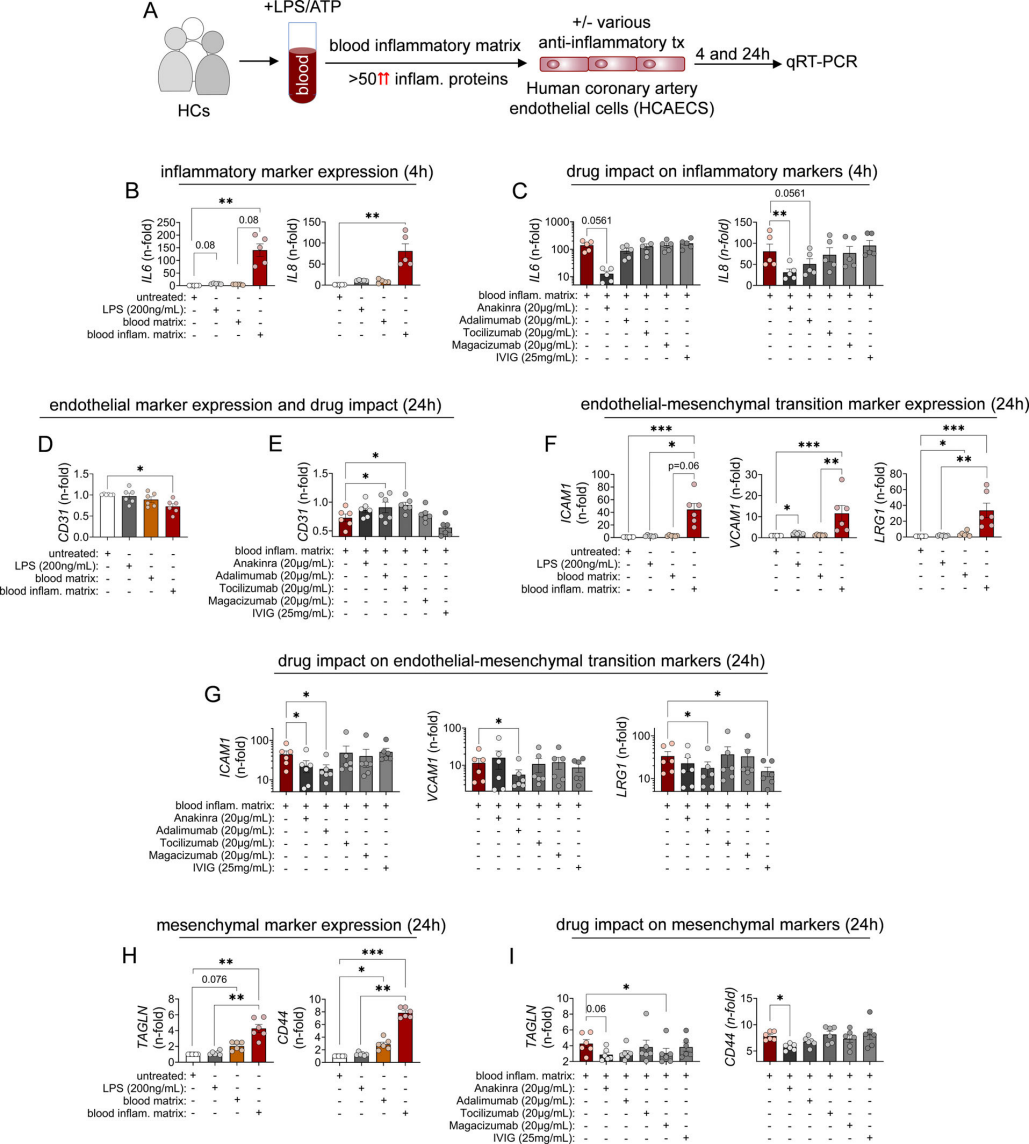
Inflammatory activation and EndMT of HCAEC induced by the blood inflammatory matrix. (A) Experimental setup. (B) Inflammatory activation of HCAECs by high‐dose LPS (200 ng/mL) compared to the blood inflammatory matrix from untreated (no LPS) or LPS (1 ng/mL) plus ATP‐treated HC blood was assessed by selected HCAEC gene expression (qRT‐PCR). (C) Inflammatory HCAEC activation was tested with or without selective IL‐1R (anakinra), TNFα (adalimumab), IL‐6R (tocilizumab), or LRG‐1 blockade (magacizumab) or IVIG treatment. (D, F, and H) EndMT of HCAECs upon 24 hours of stimulation with high‐dose LPS (200 ng/mL) compared to exposure to the blood inflammatory matrix from untreated (no LPS) or LPS (1 ng/mL) plus ATP–treated HC blood was assessed by evaluating HCAEC expression of indicated (D) endothelial, (F) transition, and (H) mesenchymal markers on gene expression level. (E, G, and I) EndMT‐related gene expression by HCAECs was tested with or without selective IL‐1R (anakinra), TNFα (adalimumab), IL‐6R (tocilizumab), or LRG‐1 blockade (magacizumab) or IVIG treatment. Data following 24 hours of stimulation are shown. Throughout each data point represents an individual experiment and donor using freshly prepared inflammatory matrix. Data were analyzed by the Friedman test for multiple paired observations. *P < 0.05; **P < 0.01; ***P < 0.001. EndMT, endothelial‐to‐mesenchymal transition; HC, healthy control; HCAEC, human coronary artery endothelial cell; IL‐1R, interleukin‐1 receptor; IVIG, intravenous immunoglobulin; KD, Kawasaki disease; LRG‐1, leucin‐rich α‐2‐glycoprotein 1; LPS, lipopolysaccharide; qRT‐PCR, quantitative reverse transcription polymerase chain reaction; TNFα, tumor necrosis factor α.

To assess whether LRG‐1 contributes to coronary artery endothelial inflammation, we stimulated HCAECs with this inflammatory matrix (freshly prepared for every experiment; Figure 2A). In our experimental approach, we benefit from the poor responsiveness of HCAECs to direct stimulation with LPS (requires >100 ng/mL)^6^; as previously demonstrated by us, lack of CD14 expression renders HCAECs unresponsive to direct LPS exposure <10 ng/mL.^6^

In the present, experiments expression of sentinel genes for inflammatory endothelial activation (*IL6* and *IL8*) was significantly up‐regulated upon cell stimulation using the blood inflammatory matrix. In contrast, stimulation with high‐dose LPS or a blood matrix without prior LPS stimulation did not affect *IL6* and *IL8* expression (Figure 2B).

Importantly, treatment of HCAECs with the LRG‐1–neutralizing antibody (magacizumab) in the context of cell stimulations using the blood inflammatory matrix did not reduce gene expression (Figure 2C). In contrast, IL‐1R blockade (anakinra) reduced *IL‐6* (median n‐fold of 151 to 11.7) and *IL8* (median n‐fold of 60.1 to 27.1) expression. Although TNF inhibition (adalimumab) still revealed modest effect on lowering *IL‐8* expression (median n‐fold of 60.1 to 36.2), IL‐6R blockade (tocilizumab) or IVIG treatment did not consistently affect *IL6* and *IL8* transcription by HCAECs when stimulated with the blood inflammatory matrix (Figure 2C).

### Inflammatory cytokine signaling, but not LRG‐1, drives EndMT of HCAECs

Our serum proteomic analysis in KD indicates processes associated with endothelial dysfunction including EndMT (Figure 1G–K). EndMT is a highly dynamic process that results in down‐regulation of endothelial‐specific surface markers (CD31) and simultaneous up‐regulation of transition (*ICAM* and *VCAM*) and mesenchymal (surface) markers (CD44, transgelin, collagen, and vimentin).^23^ Importantly, LRG‐1 expression itself is a marker of the transition process, but as a modulator of TGFβ signaling, it can also directly impact a major EndMT‐inducing pathway. Both processes have been demonstrated to be highly relevant in KD pathogenesis.^24^

Upon 24 hours of exposure to the inflammatory matrix, we observed HCAECs down‐regulate endothelial marker gene expression (*CD31*, n‐fold vs untreated = 0.75) but up‐regulate transition (*ICAM1*, n‐fold vs untreated = 43.7; *VCAM1*, n‐fold vs untreated = 10.3; *LRG1*, n‐fold vs untreated = 25.1) and mesenchymal marker (*TAGLN*, n‐fold vs untreated = 4.1; *CD44*, n‐fold vs untreated = 7.7) gene expression (Figure 2D, F, and H). Transitional marker gene expression decreased over time (48 and 72 hours), whereas mesenchymal marker gene expression peaked at 48 hours (*TAGLN*) or steadily increased (*CD44*) within the time frame of the analysis (up to 72 hours post stimulation; Figure S3A).

Drug intervention studies showed that several of the assessed genes were affected significantly (*CD31*, median n‐fold of 0.75 to 0.9 [anti‐TNF]; *ICAM1*, median n‐fold of 43.7 to 21.0 [anti–IL‐1R] or 14.7 [anti‐TNF]; *VCAM1*, median n‐fold of 10.3 to 3.5 [anti‐TNF]; *LRG1*, median n‐fold of 25.1 to 11.9 [anti‐TNF]; *CD44*, median n‐fold of 7.7 to 5.8 [anti–IL‐1R]) by either IL‐1R blockade or TNF inhibition following 24 hours of HCAEC exposure to the inflammatory matrix (Figure 2E, G, and I). Yet, compared to TNF inhibition, effects upon IL‐1R inhibition appeared more consistent over time (72 hours; Figure S3B).

In line with these results, a post hoc analysis of a microarray dataset from recombinant cytokine stimulated HCAECs^25^ (Figure S4) demonstrated a down‐regulation of genes for endothelial markers such as *TIE1*, *CD31*, *FGFR1*, and *VWF*, which could be particularly observed upon both recombinant human IL‐1β and TNF stimulation compared to treatment with type I or type II interferon (IFNβ or IFNγ) or oncostatin‐M (OSM) following 24 hours of cytokine stimulation. Except for ICAM‐1, transition markers (*ID1* and *SNAI1*) were up‐regulated upon IL‐1β but not TNF stimulation. Among mesenchymal markers, we observed a mixed picture of up‐ and down‐regulated genes following either IL‐1β or TNF stimulation, with a trend towards an increased number of consistently up‐regulated mesenchymal genes following IL‐1β stimulation. *CD44* and *CDH2* (N‐cadherin) were the strongest up‐regulated genes following both IL‐1β and TNF stimulation, which was not observed from HCAECs treated with IFNβ, IFNγ or OSM (Figure S4).

Finally, and next to gene expression analysis, in translucent microscopy we also observed, that HCAECs changed towards a spindle shaped morphology upon exposure to the blood inflammatory matrix, which can indicate EndMT. Although this spindle‐shaped cell morphology was also observed with HCAECs cultured with the blood inflammatory matrix and IL‐6R or LRG‐1 blockade or IVIGs, the cell morphology of HCAECs treated with anti‐TNF or IL‐1R blockade in the course of inflammatory stimulation remained similar to that of untouched HCAECs (Figure S5).

## DISCUSSION

In an immune‐profiling study using biosamples from a phase 2 open‐label trial of the IL‐1Ra anakinra in IVIG‐resistant patients with KD, we previously identified LRG‐1—a known modulator of endothelial activation and cardiac remodeling^20^, ^21^—as associated with IL‐1β signaling.^5^ This led us to investigate whether LRG‐1 may directly contribute to inflammatory activation of HCAECs, particularly under conditions mimicking a complex inflammatory environment as in KD.

To model this, we developed a blood inflammatory matrix, revealing elevation of 50 proteins, 19 of which overlapped with those elevated in the serum of untreated patients with KD, including LRG‐1. Stimulation of HCAECs with this matrix induced inflammatory activation, which was suppressed by IL‐1 over TNF blockade, but remained unaffected by LRG‐1 or IL‐6R inhibition or IVIG treatment. Proteomics focused on cardiovascular inflammation further revealed signatures for endothelial proliferation, angiogenesis, and EndMT as a process potentially also involving LRG‐1 signaling. Notably, HCAECs exposed to the matrix revealed features of EndMT, which for the tested parameters could be best associated with IL‐1β and TNF signaling, with no evidence for the role of LRG‐1.

Our approach used a complex, multimediator setting to explore its role. Although the blood inflammatory matrix shared significant overlap with untreated KD serum profiles, it could not fully replicate the KD serum environment. The matrix was generated using LPS and ATP, inducing a Toll‐like receptor 4/NLRP3–driven signature, which is also relevant to KD.^2^, ^6^ However, due to stimulation time and used stimulants, our setup did not fully replicate T cell–related signatures (particularly on the level of IL‐17A). Nevertheless, we believe the present model represents an advance over previous approaches using only isolated monocytes^6^ by better reflecting whole blood conditions.

With respect to the responsiveness of HCAECs to LRG‐1 signaling, HCAECs have been demonstrated to express all relevant receptors and signaling molecules.^26^ Yet, in our experiments, LRG‐1 inhibition via magacizumab showed no effect on inflammatory or EndMT gene expression.

In fact, the role of LRG‐1 in vascular remodeling is reported as variable.^27^ Although some studies suggest a proangiogenic function in mouse primary brain and human umbilical vein endothelial cells through the binding of endoglin and promotion of the Smad 1/5/8 pathway,^21^ others show a profibrotic function through the enhancement of Smad 2 or 3 phosphorylation in mouse fibroblasts and renal tubular epithelial cells.^28^, ^29^ To our knowledge, no previous studies have examined LRG‐1 effects in HCAECs, limiting comparability of our data.

In our model, potential minor LRG‐1 effects may have been masked by dominant IL‐1β and TNF responses and IL‐1 and TNF blockade significantly reduced both inflammatory activation and EndMT (TNF effect restricted to EndMT), highlighting inflammation as a key EndMT driver. Similar to TGFβ signaling, inflammatory mediators like NF‐κB, phosphatidylinositol 3‐kinase, or Notch can up‐regulate EndMT‐related transcription factors. Inflammatory EndMT is marked by simultaneous increases in inflammatory and mesenchymal markers. Combined stimuli, as present both in vivo and in our in vitro model, can amplify this transformation.^26^

The connection between IL‐1 and TNF signaling and EndMT in coronary artery endothelium may have clinical relevance. Both cytokines are already targeted therapeutically, and IL‐1β's role in KD is supported by preclinical and in vitro data.^6^, ^12^, ^13^, ^14^ Proinflammatory EndMT is implicated in the development of CAAs in KD.^8^, ^9^, ^10^ Serum samples of patients with KD also induce EndMT in human umbilical cord endothelial cells, and EndMT‐related markers are seen in the *Candida albicans* cell wall extract–induced KD mouse model.^8^, ^9^ Together, these findings suggest that targeting EndMT may mitigate vascular damage in KD, with IL‐1 and TNF blockade emerging as promising therapeutic strategies, especially in IVIG‐resistant cases.

Collectively, our data should be interpreted in the light of several limitations. Even though we attempt to mimic inflammatory complexity on the level of soluble mediators, our downstream investigations were limited to only HCAECs. This can explain a missing therapeutic effect of IVIGs in our experiments, which otherwise have proven very effective in the clinical prevention of CAAs.^1^ Beyond this, we did not study LRG‐1 impact on microvascular endothelial or smooth muscle cells as well as pericytes of the vasculature, which have previously been demonstrated as sensitive to LRG‐1 stimulation.^21^ Furthermore, predicting EndMT from blood proteomics is certainly limited, as the proteomic signature is defined by several (blood) cell types including endothelial cells. Yet, it needs to be stressed that the investigated biomaterial is the only specimen available from these very young patients, which also limits obtainable amounts (volume). These limitations prompted our approach to develop a blood inflammatory matrix to facilitate the in vitro experiments, as illustrated in the present article. Importantly, despite the potentially diverse origins of the cardiovascular proteins investigated, the KD proteomic signature still differs markedly from that of HCs.

Apart from these limitations, we believe we can show that even within a complex inflammatory environment, IL‐1 and TNF signaling prominently drive both inflammatory activation and mesenchymal transition of HCAECs. These findings reinforce the critical roles of IL‐1 and TNF in cardiovascular inflammation and remodeling and support the clinical use of their blockade in KD, particularly in IVIG‐non–responsive patients, to prevent the development of CAAs.

## AUTHOR CONTRIBUTIONS

All authors contributed to at least one of the following manuscript preparation roles: conceptualization AND/OR methodology, software, investigation, formal analysis, data curation, visualization, and validation AND drafting or reviewing/editing the final draft. As corresponding author, Dr Kessel confirms that all authors have provided the final approval of the version to be published and takes responsibility for the affirmations regarding article submission (eg, not under consideration by another journal), the integrity of the data presented, and the statements regarding compliance with institutional review board/Declaration of Helsinki requirements.

## Supporting information


**Disclosure form**.

**Appendix S1:** Supplementary Information
