# Supplementary material for: Mapping Drivers of Coronary Endothelial Activation and Endothelial‐to‐Mesenchymal Transition through Mimicking of Multimediator Inflammation in Kawasaki Disease Context
Source: ACR Open Rheumatol. 2025 Oct 12;7(10):e70110. doi: 10.1002/acr2.70110 (PMC12516014; doi:10.1002/acr2.70110)
Supplement: Supplementary file 2 — Appendix S1: Supplementary Information [file ACR2-7-e70110-s002.docx]

**Supplementary methods and data**

Mimicking multi-mediator inflammation delineates drivers of coronary artery endothelial activation and endothelial-to-mesenchymal transition in context of Kawasaki Disease

Pia Buthe^a^, Marie Charlotta Limburg^a^, Sabrina Fuehner^a^, Julia Kuehn^a^, André Jakob^b^, Isabelle Koné-Paut^c^, Stéphanie Tellier^d^, Alexandre Belot^e^, Linda Rossi-Semerano^c^, Perrine Dusser^c^, Isabelle Marie^c^, Jana Merfort^f^, Katja Masjosthusmann^f^, Claas Hinze^a^, Helmut Wittkowski^a^, Dirk Foell^a^ and Christoph Kessel^a^

^a^Department of Pediatric Rheumatology and Immunology, University Children’s Hospital Muenster, Muenster, Germany

^b^Department of Pediatric Cardiology and Pediatric Intensive Care, Ludwig-Maximilians-University, Munich, Germany

^c^Division of Pediatric Rheumatology and CEREMAIA, Bicêtre Hospital, APHP, University of Paris Saclay, Le Kremlin-Bicêtre, France

^d^Department of Pediatrics, Divisions of Nephrology, Rheumatology and Internal Medicine, University of Toulouse, Toulouse, France

^e^Departments of Pediatrics, Division of Rheumatology, Dermatology and Nephrology, University of Lyon, Lyon, France

^f^Department of General Pediatrics, University Children's Hospital Muenster, Muenster, Germany

**Corresponding author:** Dr. Christoph Kessel, Department of Pediatric Rheumatology and Immunology, University Children’s Hospital Muenster, Domagkstr. 3, 48149 Muenster, Germany; Email: [christoph.kessel@uni-muenster.de](mailto:christoph.kessel@uni-muenster.de); Phone: +49-251-83-58176; Fax: +49-251-83-58174

Supplementary Material & Methods

**Study participants and sample collection**

This is a retrospective study including serum samples from KD (n=19; n=11 used for proteomics, n=8 used in cell stimulation experiments), disease active systemic juvenile idiopathic arthritis (sJIA, n=3), multisystem inflammatory syndrome in children (MIS-C, n=3) as well as healthy pediatric controls (HC, n=16; n=10 used for proteomics, n=6 used in cell stimulation experiments; see **table 1** and **S3** for demographic and clinical details). KD sera used in proteomic studies (n=11; one sample was not available for the Olink^®^ Target 96 Cardiovascular III panel) were all collected prior to IVIG-treatment with the Department of Pediatric Cardiology, Ludwig-Maximilians-University Munich, Germany. In part, proteomic data on these samples were previously reported in different context (1). KD sera used in cell stimulations (n=8) were collected in course of the KAWAKINRA trial (Eudract Number: 2014-002715-41, ClinicalTrials.gov NCT02390596), which enrolled a total number of sixteen IVIG-resistant KD patients (resistant to 1-3 cycles of IVIG) who received daily subcutaneous anakinra injection for a total duration of 14 days (2). Clinical outcome as well as immune phenotyping related to these patients and samples have been reported previously (2, 3). For the purpose of the present study, we selected eight samples (n=4 patients) with high, and eight samples (n=4 patients) with low inflammatory profile (acc. to previously reported data) (3), both prior and after three days of daily anakinra treatment (2, 3).

SJIA patients’ sera were collected in course of active disease with the Department of Pediatric Rheumatology and Immunology at University Hospital Muenster, Germany. MIS-C patients’ (n=3) sera were collected with the Department of General Pediatrics, University Hospital Muenster, and prior to treatment. Sera of non-inflammatory controls (n=6) were collected with the Pediatric Endocrinology out-patient clinic at the Department of General Pediatrics, University Hospital Muenster.

Blood samples for blood inflammatory matrix preparation were obtained from anonymous adult healthy donors and were collected at the Institute for Transfusion Medicine and Cell Therapy, University Hospital Muenster, Germany.

Serum collection was approved by the local (Muenster: 2015-670-f-S, Munich: 17-692) ethical committees or in course of the KAWAKINRA trial (Eudract Number: 2014–002,715-41, ClinicalTrials.gov NCT02390596) (2). Throughout, parents or caregivers signed written informed consent.

**Proteomic analysis**

Serum proteomics was performed using proximity extension assays (PEA) on the Olink^®^ Target 96 platform, using Target 96 Inflammation and Target 96 Cardiovascular III panels. Blood inflammatory matrix samples were analyzed using the Olink^®^ Target 96 Inflammation panel. All samples were processed by Olink Proteomics, Uppsala, Sweden. Data were reported in normalized protein expression values (npx). NPX is an arbitrary unit in a Log2 scale calculated from inverted, normalized Ct values. All assay validation data are available on the Olink website ([www.olink.com](http://www.olink.com)).

**Multiplexed bead array assay**

Reagents for multiplexed quantification of IL-1a, IL-1β, IL-6, IL-8, IL-10, IL-18, CXCL-9 and LRG-1 were purchased from R&D Systems (Minneapolis, OH, USA). Reagents and sera or cell culture supernatants were prepared according to the manufacturer’s instructions (R&D Systems). Data acquisition and analysis was performed on a MAGPIX instrument (Merck Millipore, Darmstadt, Germany) using xPONENT v4.2 software (Luminex).

**Blood inflammatory matrix**

Leukocyte reduction system chambers (LRSC) from volunteering healthy thrombocyte donors (blood donation center, University Hospital, Muenster, Germany) were collected. The entire LRSC content was diluted 1:50 in PBS (including 1mM EDTA) to approximately adjust the white blood cell count to that of whole blood (2x10^6^/mL). Blood cell suspensions at 4ml/well (6-well suspension plates) were stimulated with LPS (*S. minnesota* R595, 1ng/mL) for a total duration of 4h at 37°C and 5% CO_2_ or left untreated. After 3.5 hours of stimulation 5mM ATP was added to all samples (incl. unstimulated) to activate the NLRP3 inflammasome. Following centrifugation (500 x g, 10min) supernatants were collected and stored at -20°C for proteomic analysis and/or immediately used for endothelial cell stimulations.

**Human coronary artery endothelial cell culture and stimulation**

Primary human coronary artery endothelial cells (HCAECs) were obtained from PELOBiotech and cultured in Endothelial Cell Growth Medium (Merck KGaA, Darmstadt, Germany). HCAECs were used from passage 3 to 12. For stimulations, 0.5 - 1 x 10^4^ cells per cm^2^ were seeded in tissue culture plates and cultured to 80% confluence. Then, cells were treated with conditioned medium containing 30% of blood inflammatory matrix or 15% of patients’ or HC serum. HCAECs exposed to blood inflammatory matrix were treated or not with recombinant IL-1Ra (anakinra), monoclonal anti-IL-1β (canakinumab), monoclonal anti-TNFa (adalimumab), monoclonal anti-IL-6R (tocilizumab), monoclonal anti-LRG-1 (magacizumab (supp. ref. 1); all drugs at 20µg/mL) or IVIG (25mg/mL) and cultured for 4 to 72h (37°C and 5% CO_2_), depending on the respective experiment. Magacizumab used in this study was a kind gift of Drs. John Greenwood and Stephen Moss (UCL, London, UK). Drug concentrations applied in experiments were in range with or exceeded peak serum/plasma levels reported from respective pharmacokinetic studies(4-6) or experimental data (magacizumab, (7) and Patent: PCT/GB2016/050439).

**RNA-Isolation and cDNA-Synthesis**

HCAECs were lyzed and RNA was isolated using the NucleoSpin RNA II isolation kit (Macherey-Nagel, Dueren, Germany) according to the manufacturer’s instructions. 1µg total RNA was directly transcribed into cDNA using RevertAid H Minus Reverse Transcriptase (Thermo Fisher Scientific, Waltham, MA USA), Oligo dT Primer (Eurofins Genomics, Ebersberg, Germany) and dNTPs (Genaxxon bioscience, Ulm, Germany) according to the reverse transcriptase protocol.

**Real Time Quantitative PCR (RT-qPCR)**

cDNA was diluted 1:5 in ddH2O and used as template. qRT-PCRs were prepared using KAPA SYBR FAST qPCR Kit (Merck) according to the manufacturer's instructions and cycling was performed on a CFX 384 Real-Time System (Bio-Rad, Feldkirchen, Germany) by the Core Facility Genomics at Muenster University medical faculty. Respective primer sequences as used in experiments are indicated in **table S1**.

**Data analysis**

Serum proteomic data were analyzed for unsupervised clustering using correlation distance and ward.D2 linkage by the pheatmap R-package and Rstudio (RStudio Team (2015). RStudio: Integrated Development for R. RStudio, Inc., Boston, MA <http://www.rstudio.com/>). Protein-protein interaction enrichment analysis was performed using the pathfindR R-package and R studio(8). Volcano plots illustrating multiple unpaired t test were generated using Graphpad Prism software (Version 10 for Windows, Graphpad Software, La Jolla, CA, USA). Data of individual serum markers were analyzed by Mann-Whitney U or t-test for paired samples (Graphpad Prism). Gene expression data were analyzed acc to fold change compared to *RPL* and *DIM* as reference genes. Data were analyzed by multi-comparison analyses using Kruskal Wallis followed by Dunn’s multiple comparison test (Graphpad Prism). Overall, *P*<0.05 was considered statistically significant.

**Table S1.** qRT-PCR oligonucleotides

| **Gene** | **forward primer (5’->3’)** | **reverse primer (5’->3’)** |
| --- | --- | --- |
| RPL | AGGTATGCTGCCCCACAAAA | TGTAGGCTTCAGACGCACGAC |
| DIM1 | AAGTGCAGTGCAACAACTCTT | CTGTGCTGGTTAGGATTTGCT |
| IL-6 | ACTCACCTCTTCAGAACGAATTG | CCATCTTTGGAAGGTTCAGGTTG |
| IL-8 | ACTGAGAGTGATTGAGAGTGGAC | AACCCTCTGCACCCAGTTTTC |
| IL-18 | TTCAACTCTCTCCTGTGAGAACA | ATGTCCTGGGACACTTCTCTG |
| ICAM1 | TGCCCTGATGGGCAGTCAAC | CCCGTTTCAGCTCCTTCTCC |
| VCAM1 | CGCAAACACTTTATGTCAATGTTG | GATTTTCGGAGCAGGAAAGC |
| CD31 | AAGTGGAGTCCAGCCGCATATC | ATGGAGCAGGACAGGTTCAGTC |
| TIE1 | ACCCGCTGTGAACAGGCCTGCAGAGA | CTTGGCACTGGCTTCCTCT |
| TAGLN | CCACAGGACTTTGATGAAGAC | ACAAAAACCCACGCAGAC |
| CD44 | TGGCACCCGCTATGTCGAG | GTAGCAGGGATTCTGTCTG |
| SNAIL | AGTTTACCTTCCAGCAGCC | AGGACAGAGTCCCAGATGAG |
| SLUG | ACTCCGAAGCCAAATGACAA | CTCTCTCTGTGGGTGTGTGT |
| LRG1 | CAGCGACCAAAAAGCCCAG | ATTTCGGCAGGTGGTTGACA |

**Table S2.** Up- and down regulated proteins in KD serum (cardiovascular panel analysis) and link with endothelial dysfunction


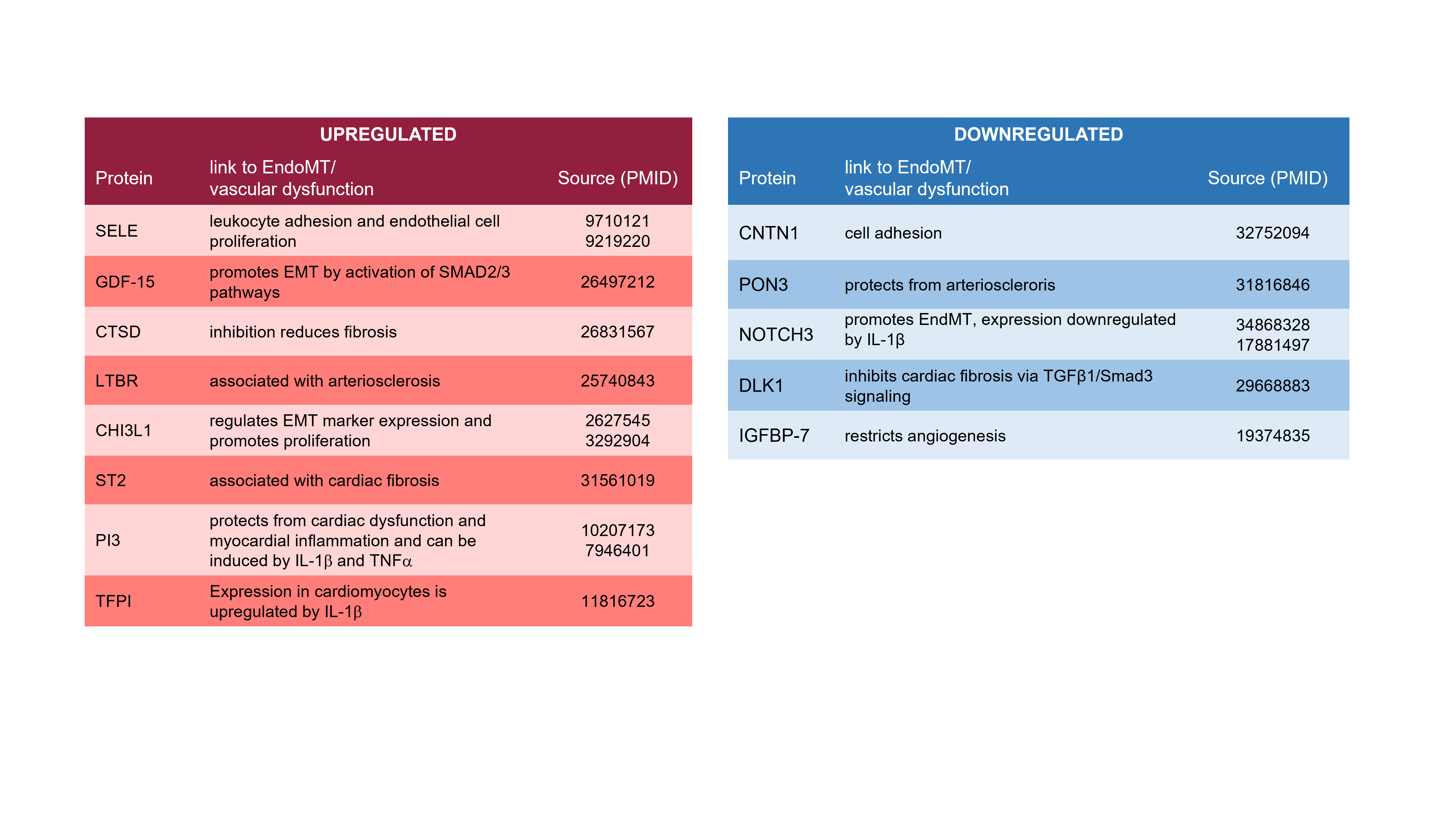


**Table S3**. Demographic and clinical data of study subjects enrolled for HCAEC stimulation using patients’/control sera (related to **Figure S1**)

|  | **HC (n=6)*** | **sJIA (n=3)** | **MIS-C (n=3)** | **KD (n=8)**  KAWAKINRA trial (supp. ref.: 8, 9) |
| --- | --- | --- | --- | --- |
| mean age (years) | 8.6 | 10.8 | 8.3 |  |
| age range (years) | 4.8 – 13.2 | 7.4 - 13.1 | 2.8 - 11.2 |  |
| sex | 3f, 3m | 2f, 1m | 2f, 1m |  |
| treatment | none | all Anakinra,  one also MTX | none | IVIG non-responder,  samples prior and after anakinra |

*non-inflammatory pediatric controls; IVIG: intravenous immunoglobulin; KD: Kawasaki Disease; MIS-C: Multisystem Inflammatory Syndrome in Children; MTX: methotrexate; sJIA: systemic juvenile idiopathic arthritis


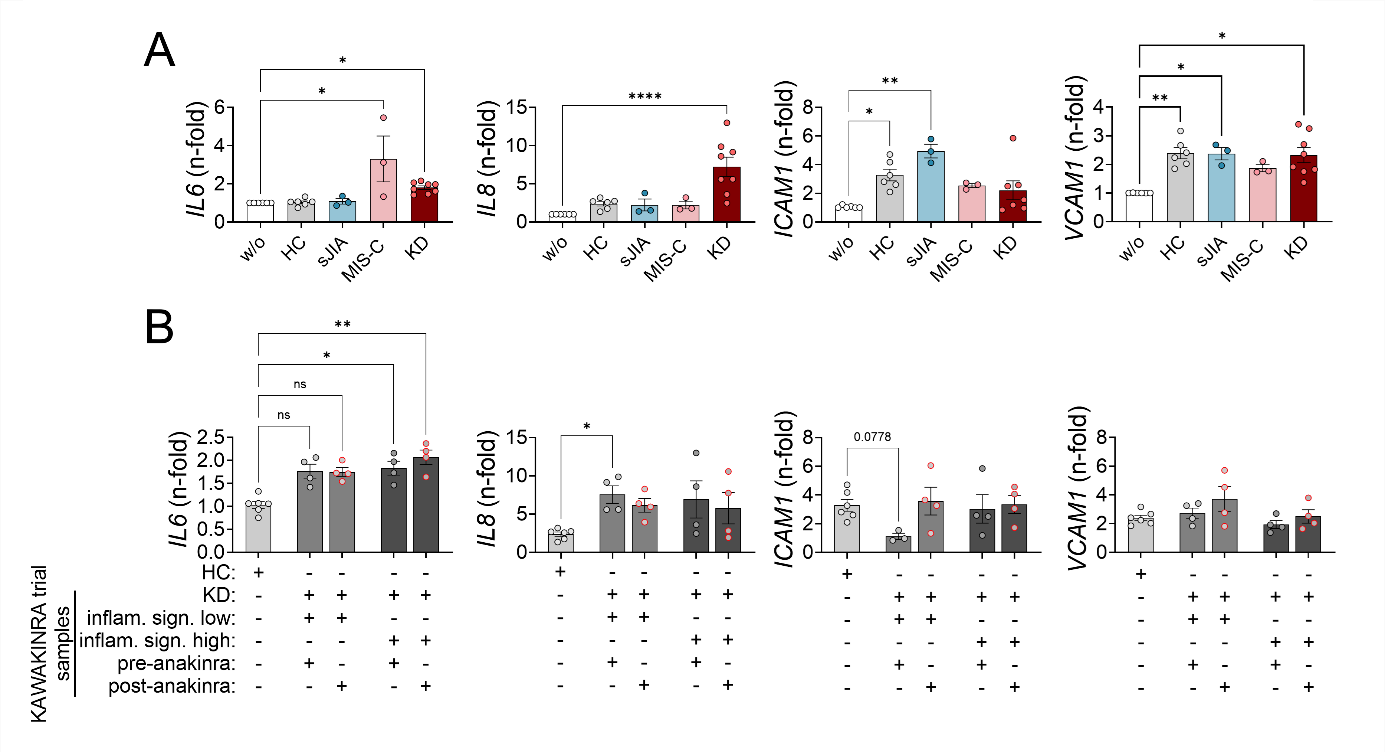


**Figure S1.** Inflammatory activation of human coronary artery endothelial cells by patients’ sera. (**A**) Inflammatory activation of HCAECs was assessed upon treatment with HC (n=6) or patients’ sera (active sJIA: n=3; active untreated MIS-C: n=3; KD, IVIG-non responders: n=8). KD samples were collected in course of the KAWAKINRA trial (supp. ref. 8). (**B**) KD samples with elevated or non-elevated inflammatory serum marker signature as reported previously (sup. ref. 9) and pre- and post-anakinra (d3) treatment were analyzed separately for impact on HCAEC stimulation. All data were analyzed by Kruskal-Wallis followed by Dunn’s post-hoc test. * = *P* < 0.05, ** = *P* < 0.01, **** = *P* < 0.0001


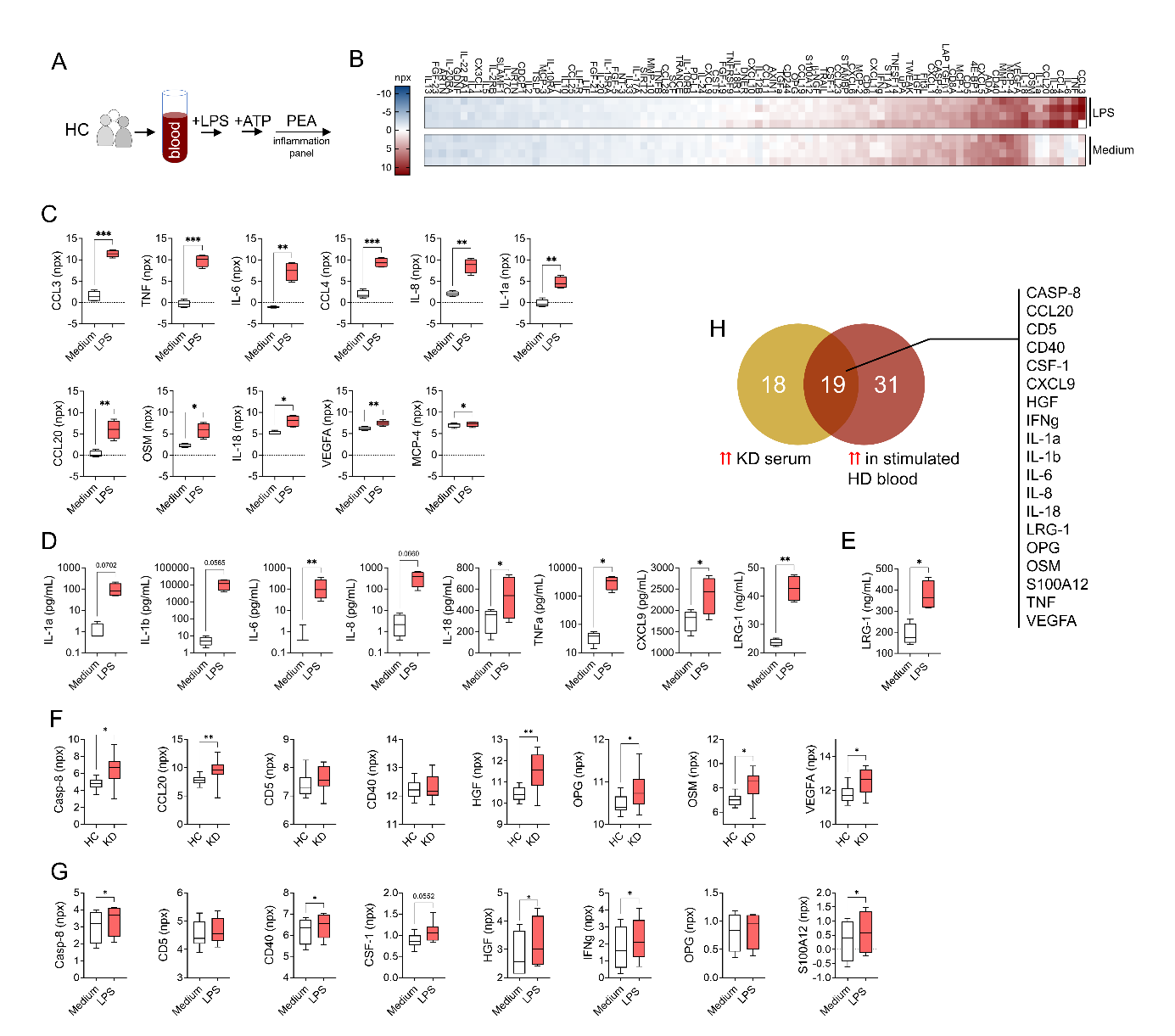


**Figure S2.** Generation of the blood inflammatory matrix and inflammatory marker profiling compared to KD patients’ sera and (related to **Figure 1A**-**F**). (**A**) HC blood was treated with LPS (3.5h) and ATP (0.5h) to generate a multi-dimensional inflammatory matrix (IM). (**B**) Representative samples (unstimulated and LPS-treated (IM) both n=4) were analyzed by PEA (Olink^Ò^ Target 96 Inflammation panel) and the head map depicts expression intensity of all analyzed markers according to npx. (**C**) Individual top upregulated markers in IM compared to untreated samples according to PEA and (**D**) additional multiplexed bead array assay (luminex) are shown. (**E**) LRG-1 quantification in blood inflammatory matrix samples at higher dilution. (**C**-**E**) Data were analyzed by paired t test. (**F**, **G**) Additional markers upregulated in (**F**) KD serum or (**G**) inflammatory matrix outside of top-upregulated. Data were analyzed by (**F**) Mann-Whitney U or (**G**) paired t test. (**H**) Venn diagram illustrating shared inflammatory mediators overexpressed in both KD sera as well as IM samples. * = *P* < 0.05, ** = *P* < 0.01, *** = *P* < 0.001, **** = *P* < 0.0001


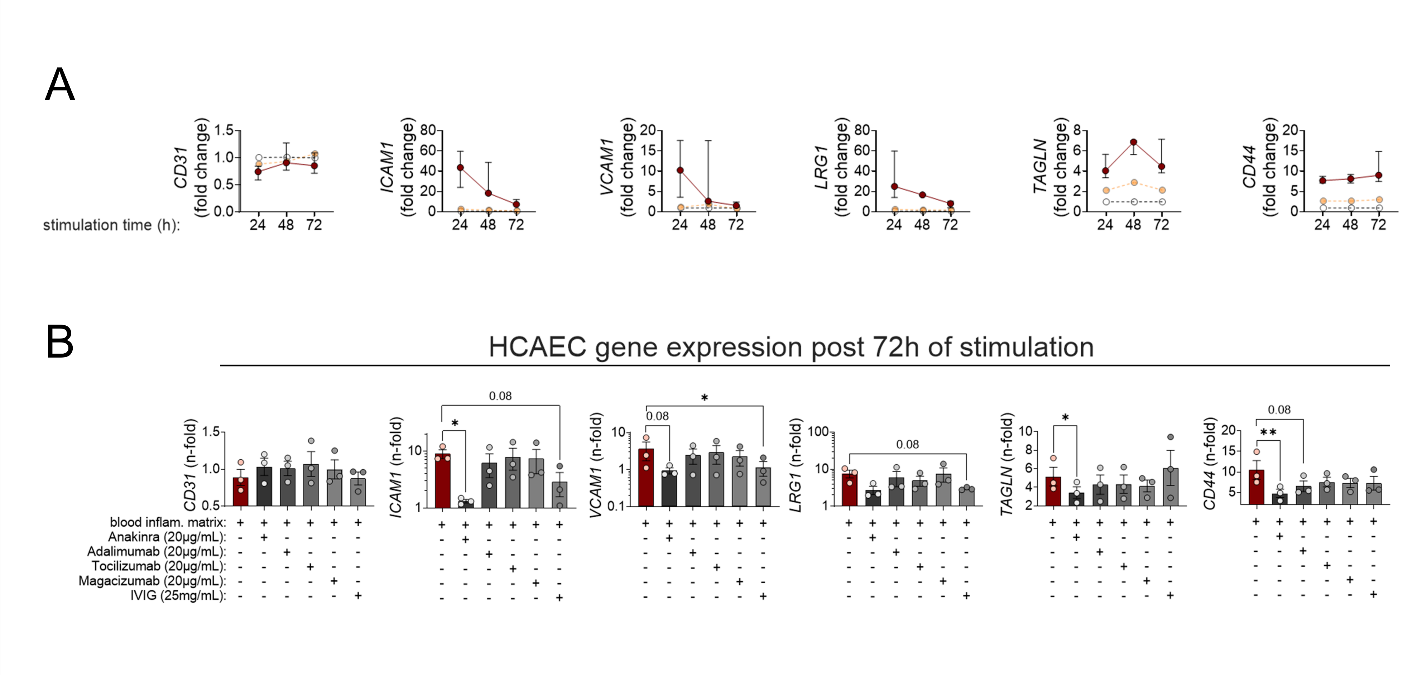


**Figure S3.** Blood inflammatory matrix induced EndMT related gene expression and drug impact. (**A**) Endothelial-to-mesenchymal transition (EndMT) of human coronary artery endothelial cells (HCAECs) upon stimulation with high-dose LPS (200ng/mL) compared to blood inflammatory matrix from untreated or LPS (1ng/mL) + ATP-treated HC blood was assessed by evaluating HCAEC-expression of indicated endothelial, transition and mesenchymal markers on gene expression level over time (24, 48 and 72h; median expression levels are shown). (**B**) EndMT-related gene expression by HCAECs was tested with or without selective IL-1R- (anakinra), TNFa- (adalimumab), IL-6R- (tocilizumab) or LRG-1-blockade (magacizumab) or IVIG-treatment. Data following 72h of stimulation are shown. Each data point represents an individual experiment and donor using freshly prepared blood inflammatory matrix for cell stimulations. Data were analyzed by Friedman test for multiple paired observations. * = *P* < 0.05, ** = *P* < 0.01


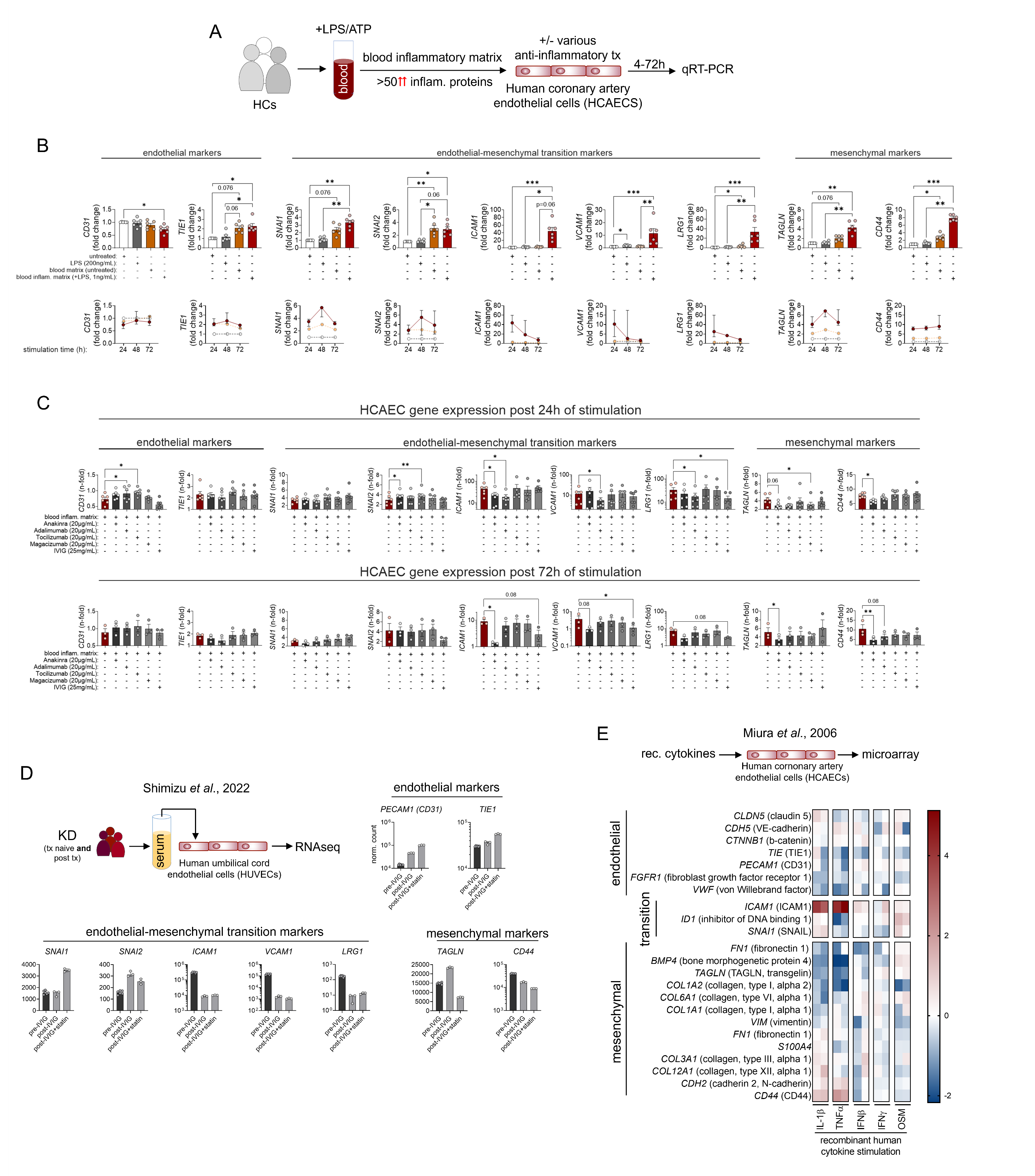


**Figure S4.** Post-hoc analysis of microarray data from recombinant cytokine stimulated HCAECs (9) for expression of indicated genes at 24h post stimulation.


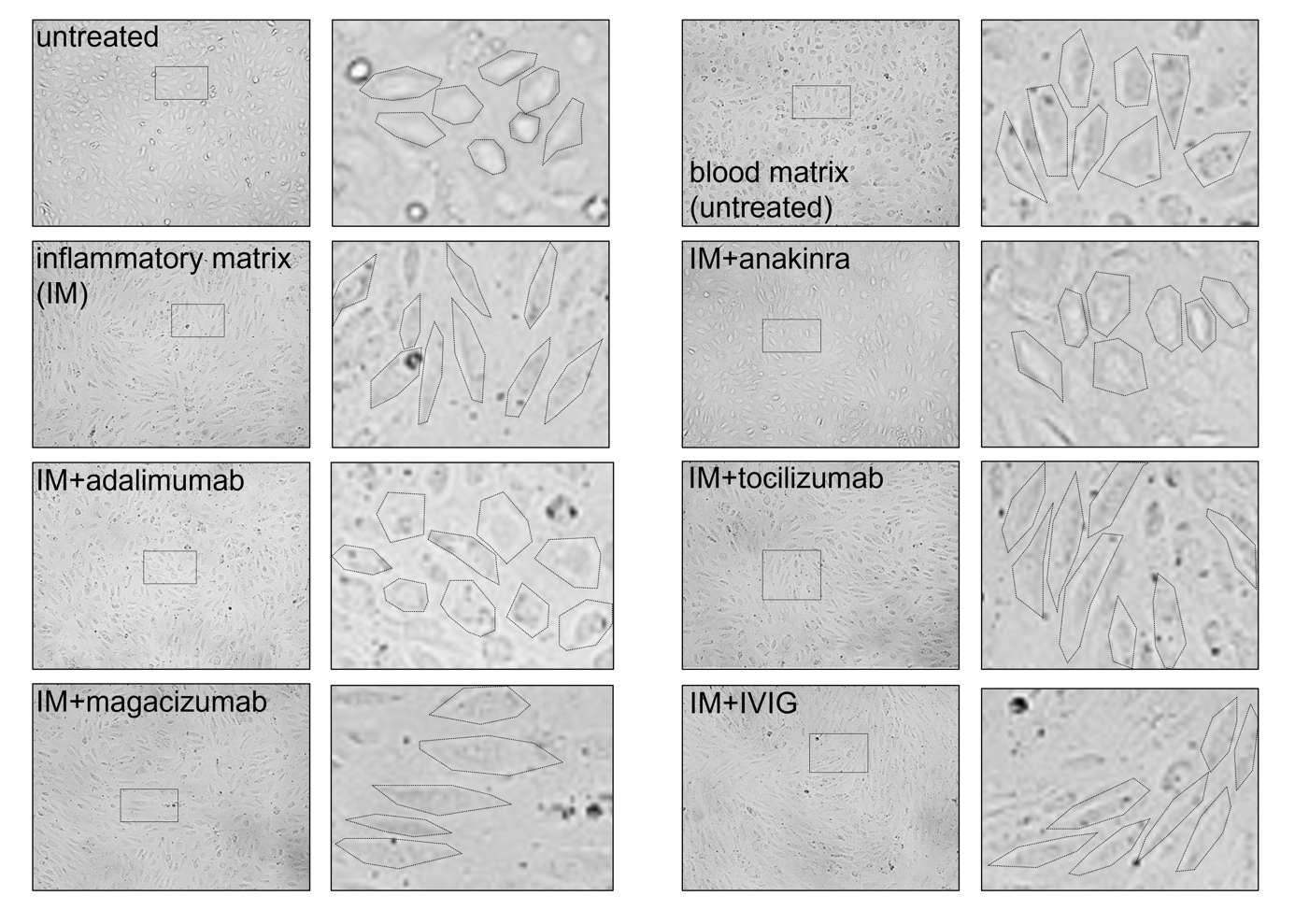


**Figure S5**. Transmitted light microscopy images illustrate changes in cell morphology with and without exposure to blood inflammatory matrix, and in absence or presence of indicated drugs. Exemplary cell morphology is enhanced by drawn contour lines in side-by-side enlarged frames of each microscopy image.

**Supplementary references**

1. Reiter A, Verweyen EL, Queste E, Fuehner S, Jakob A, Masjosthusmann K, et al. Proteomic mapping identifies serum marker signatures associated with MIS-C specific hyperinflammation and cardiovascular manifestation. Clin Immunol. 2024;264:110237.

2. Kone-Paut I, Tellier S, Belot A, Brochard K, Guitton C, Marie I, et al. Phase II Open-Label Study of Anakinra in Intravenous Immunoglobulin-Resistant Kawasaki Disease. Arthritis Rheumatol. 2021;73(1):151-61.

3. Kessel C, Kone-Paut I, Tellier S, Belot A, Masjosthusmann K, Wittkowski H, et al. An Immunological Axis Involving Interleukin 1beta and Leucine-Rich-alpha2-Glycoprotein Reflects Therapeutic Response of Children with Kawasaki Disease: Implications from the KAWAKINRA Trial. J Clin Immunol. 2022;42(6):1330-41.

4. Kuemmerle-Deschner JB, Ramos E, Blank N, Roesler J, Felix SD, Jung T, et al. Canakinumab (ACZ885, a fully human IgG1 anti-IL-1beta mAb) induces sustained remission in pediatric patients with cryopyrin-associated periodic syndrome (CAPS). Arthritis Res Ther. 2011;13(1):R34.

5. Mallalieu NL, Wimalasundera S, Hsu JC, Douglass W, Wells C, Penades IC, et al. Intravenous dosing of tocilizumab in patients younger than two years of age with systemic juvenile idiopathic arthritis: results from an open-label phase 1 clinical trial. Pediatr Rheumatol. 2019;17(1).

6. Yang J, Jain S, Capparelli EV, Best BM, Son MB, Baker A, et al. Anakinra Treatment in Patients with Acute Kawasaki Disease with Coronary Artery Aneurysms: A Phase I/IIa Trial. J Pediatr. 2022;243:173-80 e8.

7. Kallenberg D, Tripathi V, Javaid, F, Pilotti, C, George, J, Davis, S, et al. A Humanized Antibody against LRG1 that Inhibits Angiogenesis and Reduces Retinal Vascular Leakage. bioRxiv, 2021; doi.org/10.1101/2020.07.25.2181492021.

8. Ulgen E, Ozisik O, Sezerman OU. pathfindR: An R Package for Comprehensive Identification of Enriched Pathways in Omics Data Through Active Subnetworks. Front Genet. 2019;10.

9. Miura A, Honma R, Togashi T, Yanagisawa Y, Ito E, Imai J, et al. Differential responses of normal human coronary artery endothelial cells against multiple cytokines comparatively assessed by gene expression profiles. FEBS letters. 2006;580(30):6871-9.
